# Combined Blood Indexes of Systemic Inflammation as a Mirror to Admission to Intensive Care Unit in COVID-19 Patients: A Multicentric Study

**DOI:** 10.1007/s44197-021-00021-5

**Published:** 2021-12-13

**Authors:** Dina Ali Hamad, Mai Mostafa Aly, Marwa Ahmed Abdelhameid, Shimaa Abdalla Ahmed, Asmaa Salah Shaltout, Alaa Eldin Abdel-Moniem, Ahmed Mohamed Reda Ragheb, Mohammed Nahed Attia, Taghreed Sayed Meshref

**Affiliations:** 1 Critical Care Unit, Department of Internal Medicine, Faculty of Medicine, Assiut University, Assiut, Egypt; 2 Clinical Hematology Unit, Department of Internal Medicine, Faculty of Medicine, Assiut University, Assiut, Egypt; 3 Department of Internal Medicine, Faculty of Medicine, Aswan University, Aswan, Egypt; 4 Department of Internal Medicine, Faculty of Medicine, Qena University, Qena, Egypt; 5 Department of Medical Microbiology & Immunology, Faculty of Medicine, Assiut University, Assiut, Egypt; 6 Department of Anesthesiology, Faculty of Medicine, Aswan University, Aswan, Egypt; 7 Department of Oral and Maxillofacial Surgery, Faculty of Dentistry, Assiut University, Assiut, Egypt

**Keywords:** COVID-19, AISI, SII, Intensive care unit, Egypt

## Abstract

**Background:**

The Coronavirus 2019 is a pandemic that has spread worldwide, threatening human health. The main cause of death in patients with COVID-19 is a systemic pro-inflammatory mechanism that quickly progresses to acute respiratory distress syndrome. Hematological ratios as affordable indicators of inflammatory response were studied in COVID-19 patients. The study aimed to study the importance of the blood cell indexes of the systemic inflammatory response, as the Aggregate Index of Systemic Inflammation (AISI), neutrophils lymphocyte to platelet ratio (NLPR), systemic immune-inflammation index (SII) and, systemic inflammation response index (SIRI) in predicting intensive care unit (ICU) admission of COVID-19 patients.

**Methods:**

495 COVID-19 patients managed in four tertiary centers; divided into non-ICU and ICU groups.

**Results:**

Total leucocyte count (TLC), AISI, NLPR, SII, and SIRI were more elevated in the ICU group (*P* < 0.001 for all except AMC *P* = 0.006), while this group had less absolute lymphocyte count (ALC) (*P* = 0.047).

We estimated the optimal cut-off values of the hematological ratio; AISI (729), NLPR (0.0195), SII (1346), and SIRI (2.5). SII had the highest specificity (95.6%), while NLPR had the highest sensitivity (61.3%). Age, AISI, CRP, D-dimer, and oxygen aid were the independent predictors for ICU admission in COVID-19 in multivariate logistic regression.

**Conclusion:**

AISI is a predictor for severity and ICU admission in COVID-19 patients, SII is a predictor of survival, while NLPR and SIRI have an additive role that needs further evaluation.

**Supplementary Information:**

The online version contains supplementary material available at 10.1007/s44197-021-00021-5.

## Introduction

COVID-19 continued to spread, invading about 223 countries worldwide [1]. Epidemiologists believe COVID-19 is here to stay, and annual fluctuations in infection could exist by 2025 and beyond [2]. Low- and middle-income countries are expected to suffer longer than other countries, probably as the future of this virus is affected by social and economic status. Almost 20% of hospitalized patients need ICU admission, with a mortality rate reaching 61.5% in some regions [3]. Also, COVID-19 patients admitted to ICU have a twofold greater risk of thrombotic complications than non-ICU patients [4].These facts force us to investigate cheap and informative methods to detect the risky population who may need intensive care unit (ICU) admission.

Blood indexes were extensively studied in COVID-19 patients, such as the neutrophil to lymphocyte ratio (NLR) [5], derived NLR [5], platelet to lymphocyte ratio (PLR) [6], and lymphocyte to monocyte ratio (LMR) [7]. Red cell distribution width (RDW) was another important blood index that was studied in COVID-19 patients. A previous meta-analysis showed that elevated RDW is associated with adverse outcomes in COVID-19 patients [8].Other indexes, which include three or more blood values, are less studied with COVID-19, such as Aggregate Index of Systemic Inflammation (AISI), neutrophils lymphocyte to platelet ratio (NLPR), systemic immune-inflammation index (SII) and, systemic inflammation response index (SIRI).

AISI (neutrophils*monocytes* platelets/lymphocytes) is a unique parameter in neoplastic conditions as non-small-cell lung cancer [9]. Very few studies evaluated its link to COVID-19 victims; none of them was linked to severity [10]. Among thousands of researches on COVID-19, only one detected the predictive value of NLPR (neutrophil to lymphocyte*platelet ratio) in infected patients [11].

SII, which depends on the number of lymphocytes, neutrophils, and platelets, is an indicator associated with inflammation and can reflect the immune and inflammatory state [12]. Some studies have identified a clear correlation between SII and prognosis in malignancy and inflammatory conditions [13, 14]. However, there are limited data about the benefit of SII in assessing the prognosis in COVID-19 patients.

It is observed that SIRI (neutrophil*platelet to lymphocyte ratio) is correlated with clinical outcomes and predicts the survival of gastric [15] and breast cancers [16]. This efficient parameter can properly represent the inflammatory and immune status balance with datasets in COVID-19 patients [10].

In this research, we aimed to study these combined blood cell indexes of systemic inflammation, the association between AISI, NLPR, SII, and SIRI and the need for ICU admission hoping to contribute to clinical practice.

## Methods

### Study Design

This retrospective study included 495 COVID-19 patients admitted in four tertiary hospitals in Egypt (Assiut University Hospital, El Rajhi Hospital, Aswan University Hospital, Qena University Hospital) during July 2020. COVID-19 diagnosis based on WHO interim guidelines [17]. All patients were treated according to recommendations from the World Health Organization (WHO) [17].

### Data Collection

Data of the patients were collected from the hospital records following the patients' consents to share the data and the authorization of the local research ethics committee according to the Declaration of Helsinki. It included age, sex, history of smoking, associated comorbidities [diabetes mellitus (DM), hypertension (HTN), cardiovascular disease (CVD), chronic obstructive pulmonary disease (COPD)], vital signs at admission, duration of hospitalization, treatment (steroid and oxygen supply either by low flow oxygen supplementation (nasal cannula, facial masks, or non-rebreather facial masks) or high-flow oxygen supplementation (high-flow nasal cannula, continuous positive ventilation pressure (CPAP) or mechanical ventilation),and outcomes.

Investigations included complete blood picture (CBC), d-dimer, ferritin and C reactive protein (CRP). Blood Indexes of Systemic Inflammation were calculated from CBC according to the following equations: AISI = neutrophil*platelet* monocyte to lymphocyte ratio, NLPR = neutrophil to lymphocyte*platelet ratio, SII = neutrophil*platelet to lymphocyte ratio and, SIRI = neutrophil* monocyte to lymphocyte ratio. All collected laboratory results were recorded at day of admission.

Computed tomography (CT) of the chest of the patients was classified into specific findings suggestive of COVID19 infection as bilateral or unilateral multifocal ground-glass opacities that classically predominate in the peripheral, posterior, and basal part of the lungs or other less specific findings. CORADS classification was scored from very low or CO-RADS 1 to very high or CO-RADS 6 based on the CT findings.

Patients were grouped according to admission site into (1) ICU group: patients with severe presentations who were admitted to ICU. (2) Non- ICU group: patients with less severe presentations admitted to the ward. The decision regarding ICU admission depended on the Modified National Early Warning Score (Modified NEWS) for COVID-19 patients [18]. Supplementary Table 1.

### Statistical Analysis

Continuous variables were either expressed as suitable means and standard deviations or medians and interquartile ranges. Categorical variables were presented as the counts and percentages in each category. We grouped the patients into ICU and non-ICU groups. Wilcoxon rank-sum analysis was employed to continuous variables, and for categorical variables, chi-square and Fisher's exact tests were used. By applying the receiver operating curve (ROC) analysis, we determined the optimal cut-off values of the continuous AISI, NLPR, SII, and SIRI. Kaplan–Meier curves of AISI, NLPR, SII, and SIRI were used to determine the survival time of COVID-19 patients. As common indicators to assess relative risk, hazard risk (HR) and 95% confidence interval (CI) were used. Binary logistic regression analysis was performed to assess the effect of age, gender, and all other relevant factors. *P* < 0.05 was considered to be statistically significant. All these data analyses were conducted with the software SPSS 170 (SPSS Inc, Chicago, USA).

## Results

### Clinical, Laboratory, and Imaging Characteristics of the Studied Population

Demographic and baseline data of the studied cohort are shown in (Table 1). This study populations were divided into two groups: non-ICU (*n* = 185; 37.4%) and ICU (*n* = 310; 62.6%). Older patients were more in the ICU group in comparison to the non-ICU group (median = 58 vs. 33 years) (*P* < 0.001). Most of the patients were males in both groups (*P* = 0.41).

**Table 1:** Demographic data of the studied population

**Variant**	Group		*P*-value
	Non-ICU *n* = 185	CCU *n* = 310	
Age/years(median)	33	58	< 0.001*
Sex /males *n* (%)	91(49.2%)	181(58.4%)	0.013**
Smoking *n* (%)	37(20.0%)	65(21.0%)	0.754**
Comorbidities *n* (%)			
DM	11(6.0%)	81(26.1%)	< 0.001**
HTN	14(7.6%)	113(36.5%)	< 0.001**
CVD	9(4.9%)	85(27.4%)	< 0.001**
COPD	5(2.7%)	35(11.3%)	< 0.001***
Laboratory data (mean ± SD)			
HB (g/dL)	12.14 ± 2.54	11.67 ± 2.55	0.021*
Platelets (× 10^9^/L)	208.03 ± 103.65	256.55 ± 130.87	< 0.001*
TLC (× 10^9^/L)	5.31 ± 3.57	10.79 ± 7.06	< 0.001*
ANC (× 10^9^/L)	2.96 ± 2.55	8.40 ± 5.88	< 0.001*
ALC (× 10^9^/L)	1.72 ± 1.22	1.56 ± 1.06	0.047*
AMC (× 10^9^/L)	0.41 ± 0.44	0.69 ± 0.91	0.006*
NLR	2.04 ± 1.68	7.40 ± 6.61	< 0.001*
AISI	256.38 ± 505.47	1318.81 ± 1948.85	< 0.001*
NLPR	0.01 ± 0.02	0.04 ± 0.05	< 0.001*
SII	492.29 ± 804.49	2016.29 ± 2162.88	< 0.001*
SIRI	1.05 ± 1.60	4.91 ± 7.60	< 0.001*
CRP (mg/l)	33.30 ± 44.74	96.82 ± 120.29	< 0.001*
Ferritin (mcg/ml)	241.74 ± 221.54	235.55 ± 237.43	0.175*
D-dimer (mcg/ml)	0.63 ± 0.46	4.57 ± 2.92	< 0.001*
MSCT *n* (%)			
Bilateral affection or multiple unilateral affection	168 (90.8%)	310 (100.0%)	< 0.001***
Oxygen support *n* (%)			
Any oxygen aids other than mechanical ventilation	27 (14.6%)	112 (36.1%)	< 0.001**
Mechanical ventilation	0 (0.0%)	99 (67.8)	< 0.001**
Outcome *n* (%)			
Death	13 (7%)	120 (38.7%)	< 0.001**

Bold indicates *P* value < 0.05 are statistically significant

*AISI* Aggregate Index of Systemic Inflammation, *ALC* absolute lymphocytic count, *ANC* absolute monocyte count, *ANC* absolute neutrophil count, *COPD* chronic obstructive pulmonary disease, *CRP* C-reactive protein, *CVD* Cardio Vascular Disease, *DM* diabetes mellitus, *HB* hemoglobin, *HTN* hypertension, *LMR* lymphocyte to monocyte ratio, *MSCT* multi-slice computed tomography, *NLPR* neutrophils lymphocyte to platelet ratio, *NLR* neutrophil to lymphocyte ratio, *SII* systemic immune-inflammation index, *SIRI* systemic inflammation response index, *WBCs* white blood cells

*Mann–Whitney test

*Chi-square test

***Fisher`s Exact test

Regarding the associated comorbidities, most ICU patients had D.M., HTN, CVD, and COPD compared to the non-ICU group (*P* < 0.001).

Regarding the hematological parameters, hemoglobin level was higher in the non-ICU group vs. ICU group, with a significant difference (*P* = 0.021). On the other side, platelets (PLT) were lower in the non-ICU group (*P* < 0.001).

The total leucocyte count (TLC), absolute neutrophil count (ANC), and, NLR were higher in the ICU group with a statistically significant difference (*P* < 0.001). Absolute monocyte count (AMC) was also statistically significantly higher in the ICU group (*P* = 0.006). In comparison, absolute lymphocyte count (ALC) was lower in the ICU than in the non-ICU group, with a significant difference (*P* = 0.047).

The AISI, NLPR, SII, and SIRI were higher in the ICU group with statistically significant difference (*P* < 0.001).

Inflammatory markers in our study showed that higher CRP and d-dimer were found in the ICU group (*P* < 0.001), while more elevated ferritin tended to be in the non-ICU group (*P* = 0.175).

MSCT imaging showed more extensive lesions (either bilateral lesions or multiple unilateral lesions) in all patients of the ICU group compared to 90.8% of the non-ICU group (*P* < 0.001).

The oxygen aid, either low flow as simple facial masks, nasal cannula or non-rebreather facial masks or high flow as continuous positive ventilation pressure (CPAP), was given to 87.1% of ICU group patients compared to 14.6% of non-ICU group (*P* < 0.001). One-third (36.1%) of ICU patients were mechanically ventilated.

Death occurred in 7% vs. 38.7% in non-ICU and ICU groups, respectively, with a significant difference between both groups (P < 0.001).

### ROC Curves to Detect the Optimum Cut-Off Values of Hematological Indexes to Differentiate ICU from Non-ICU COVID-19 Infection

**Fig. 1:**
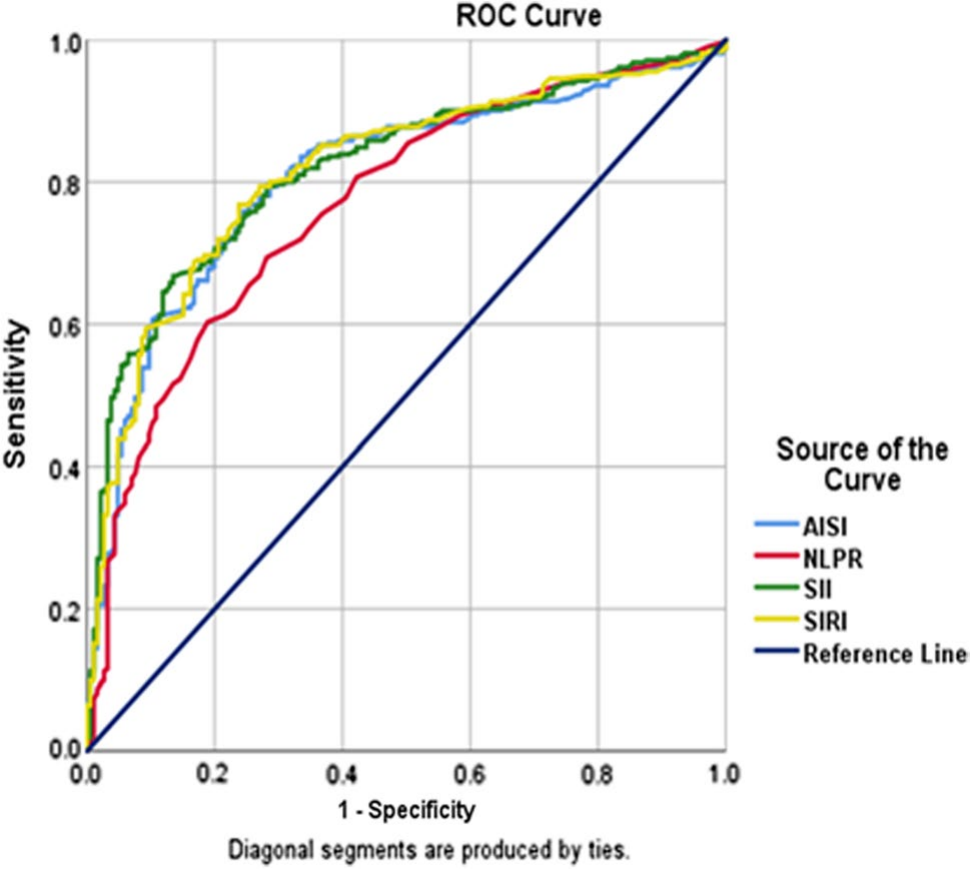
ROC curves to detect the optimum cut-off values of hematological ratios, AISI; Aggregate Index of Systemic Inflammation (> 729); NLPR; neutrophils lymphocyte to platelet ratio (> 0.0195); SII; systemic immune-inflammation index (> 1346) and SIRI; systemic inflammation response index (> 2.5)

We analyzed the optimal cut-off values of AISI, NLPR, SII, and SIRI calculated by the ROC analysis and presented in (Fig. 1). The area under the curve (AUC) of AISI, NLPR, SII, and SIRI was 0.807, 0.768, 0.819, and 0.815. The optimal cut-off values were 728, 0.0195, 1346, and 2.5 for AISI, NLPR, SII, and SIRI, respectively. SII had the highest specificity (95.6%) followed by AISI, then SIRI, then NLPR (92.8%, 91.9%, and 80.9% respectively), while the highest sensitivity was in favor of NLPR (61.3), then SIRI, then AISI, and last SII (59.4%, 51.7%, 50.9% respectively) (Table 2).

**Table 2:** Area under curve (AUC) of AISI, NLPR, SII and SIRI

	AUC	*P*- value	95% CI		Cut off point	Sensitivity (%)	Specificity (%)
			Lower	Upper			
AISI	0.807	** < 0.001**	0.767	0.846	> 729	51.7	91.9
NLPR	0.768	** < 0.001**	0.725	0.810	> 0.0195	61.3	80.9
SII	0.819	** < 0.001**	0.782	0.856	> 1346	50.9	95.6
SIRI	0.815	** < 0.001**	0.777	0.854	> 2.5	59.4	92.8

The test variable(s): AISI, NLPR, SII and SIRI had at least one tie between the positive actual state group and the negative actual state group.

*AISI* Aggregate Index of Systemic Inflammation, *AUC* area under curve*, CI* confidence interval, *NLPR* neutrophils lymphocyte to platelet ratio, *SII* systemic immune-inflammation index,* SIRI* systemic inflammation response index, *PV* predictive value

### Kaplan–Meier Curves of AISI, NLPR, SII, and SIRI to Determine the Survival Time of COVID-19 Patients

The estimated mean time until death was 21.35 days for non- ICU, and 17.75 days for the ICU group (*P* < 0.001).

**Fig. 2:**
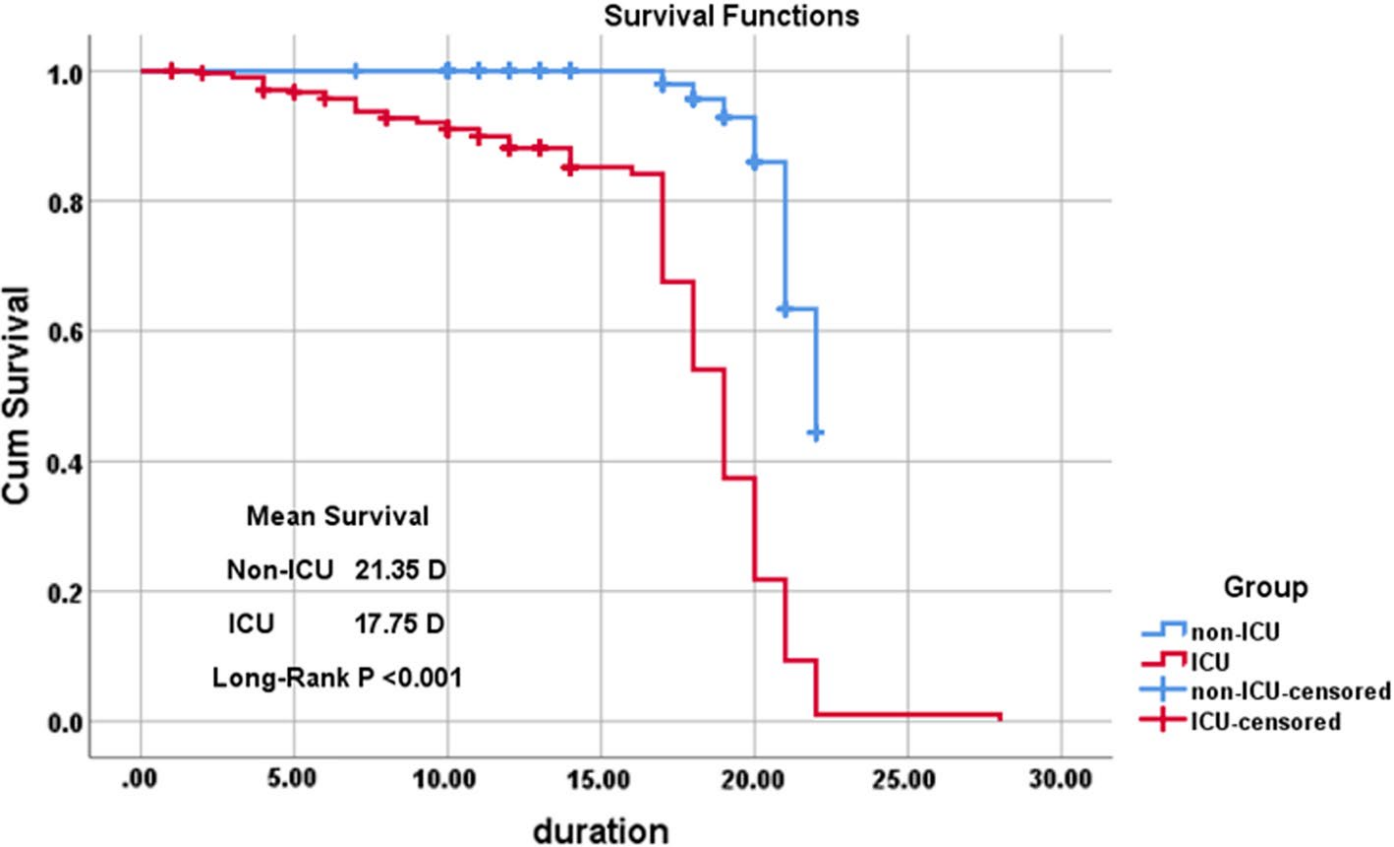
Survival rate using Kaplan–Meier in the studied groups; (non-ICU and ICU groups) showing mean survival 21.35 days in non-ICU vs. 17.75 days in ICU patients (P < 0.001)

Figure 2 shows that the survival probability is lower for ICU patients at all-time points, so they are less likely to survive.

**Fig. 3:**
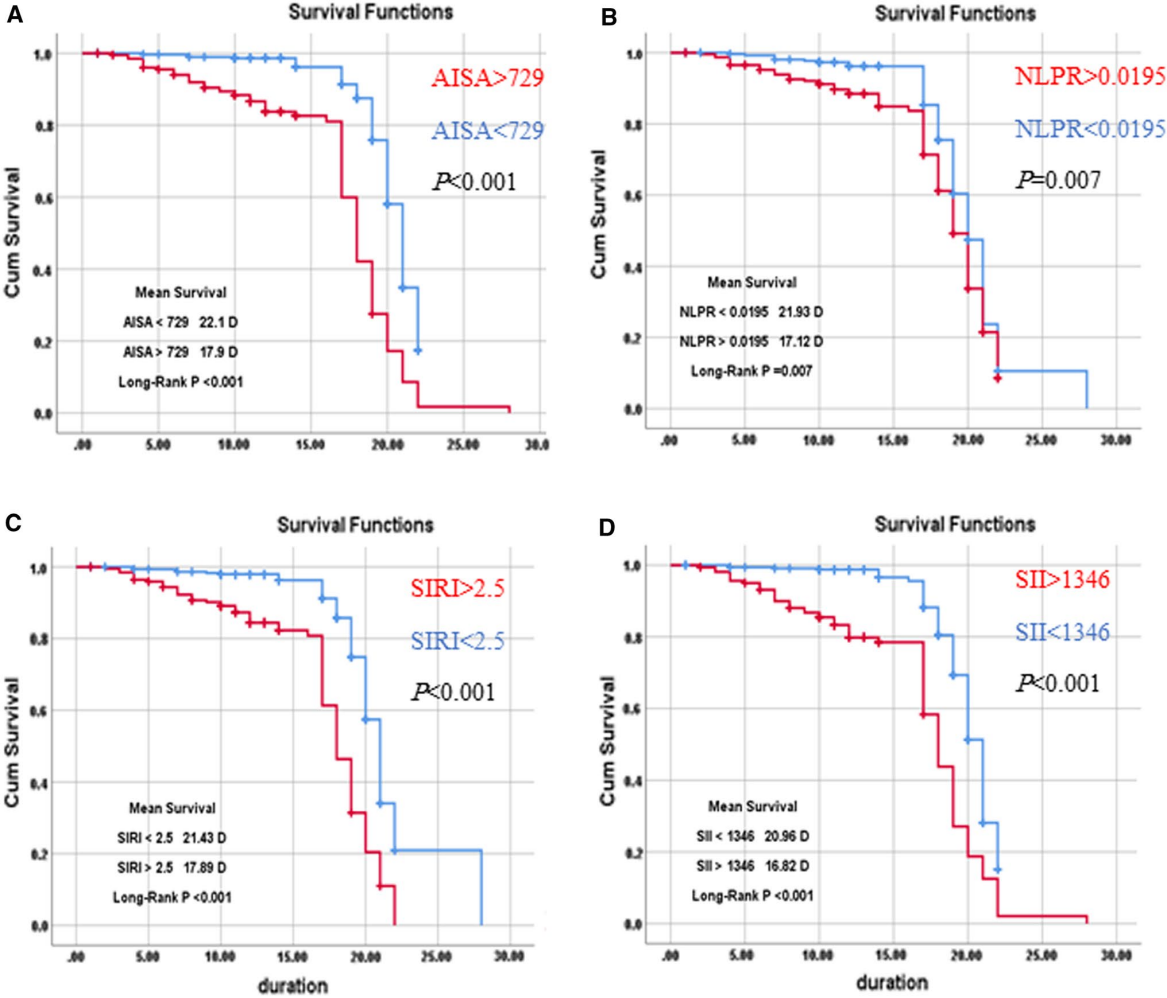
Survival analysis using Kaplan–Meier curves according to the studied blood indexes of systemic inflammation: **A** AISI > 729 vs AISI < 729; *P* < 0.001, **B** NLRP > 0.0195 vs. NLRP < 0.0195; *P* = 0.007 **C** SIRI > 2,5 vs. SIRI < 2.5; *P* < 0.001 and (D)SII > 1346 vs. SII < 1346; *P* < 0.001. AISI, Aggregate Index of Systemic Inflammation; NLPR, neutrophils lymphocyte to platelet ratio; SII, systemic immune-inflammation index; SIRI, systemic inflammation response index

Mean survival time was estimated according to AISI (estimated mean time until death is 22.1 days for victims with AISI < 729 and 17.9 days for those with AISI > 729, NLPR (The estimated mean time until death is 21.93 days for patients with NLPR < 0.0195 and 17.12 days for patients with NLPR > 0.0195), SII (the estimated mean time until death is 20.96 days if SII < 1346 and 16.82 days if SII > 1346), and SIRI (The estimated mean time until death is 21.43 days if SIRI < 2.5 and 17.89 days if SIRI > 2.5). So, it is obvious that those patients with AISI > 729, NLPR > 0.0195, SII > 1346, and SIRI > 2.5 are less likely to survive (Fig. 3).

### Identification of Possible Predictors of ICU Admission in COVID-19 Cases

To determine the impact of the above indicators on patients’ prognosis with COVID-19, we also performed Kaplan–Meier survival analysis and COX regression analysis to explore the possible independent predictors for ICU admission of COVID-19. Further univariate and multivariate COX regression analysis showed that AISI(HR 1.000, 95% CI 1.000–1.002), NLPR(HR 1.647, 95% CI 0.280–9.681), SII(HR 1.001, 95% CI 1.001–1.003) and SIRI (HR 1.015, 95% CI 1.003–1.027) were identified by univariate Cox regression but only raised SII(HR 1.004, 95% CI 1.000–1.006) was the independent factor affecting the recovery and discharge of patients with COVID-19 in multivariate analysis.

Age, male sex, DM, HTN, cardiovascular diseases, COPD, NLR, AISI, NLPR, SII, SIRI, CRP, d-dimer, steroid, oxygen aids, and mechanical ventilation were consistent with COVID-19 disease severity in the univariate logistic regression study. We integrated all the above parameters with statistical significance in the univariate analysis for in-depth analysis into the multivariate logistic regression model. In the multivariate logistic regression model, considering the likelihood of overfitting, we assumed a stepwise forward method for logistic regression analysis to decrease the number of independent variables entering the model, aiming to decrease the probability of overfitting the model. The results showed that the early independent predictors for ICU entry in COVID-19 were age, AISI, CRP, D-dimer, and oxygen support following admission (Table 3).

**Table 3:** Univariate and multivariate logistic regression analysis of risk factors associated with ICU admission in COVID-1

	Univariable Odds ratio	95% CI		*P*-value	Multivariable Odds ratio	95% CI		P value
		Lower	Upper			Lower	Upper	
Age	1.072	1.057	1.087	< 0.001	1.054	1.005	1.078	0.048
Sex (male vs. female)	1.601	1.104	2.321	0.013	1.752	0.790	3.338	0.196
DM (yes vs. no)	5.563	2.875	10.763	< 0.001	1.023	0.345	1.893	0.461
HTN (yes vs. no)	6.965	3.853	12.590	< 0.001	1.496	0.718	5.215	0.982
Cardiovascular diseases (yes vs no)	7.388	3.615	15.099	< 0.001	3.058	0.921	8.878	0.544
COPD (yes VS no)	4.599	1.768	11.959	0.002	3.559	1.828	8.969	0.765
AISI	1.004	1.003	1.005	< 0.001	1.003	1.001	1.005	0.003
NLPR	1.001	0.0076	1.001	< 0.001	1.000	0.999	1.002	0.843
SII	1.002	1.001	1.002	< 0.001	1.008	0.626	1.967	0.768
SIRI	1.959	1.668	2.301	< 0.001	1.014	1.004	1.024	0.789
CRP	1.021	1.016	1.027	< 0.001	1.010	1.002	1.018	0.012
Ferritin	1.000	0.999	1.001	0.773				
D-dimer	25.207	13.327	47.680	< 0.001	20.101	8.005	50.478	< 0.001

**Fig. 4:**
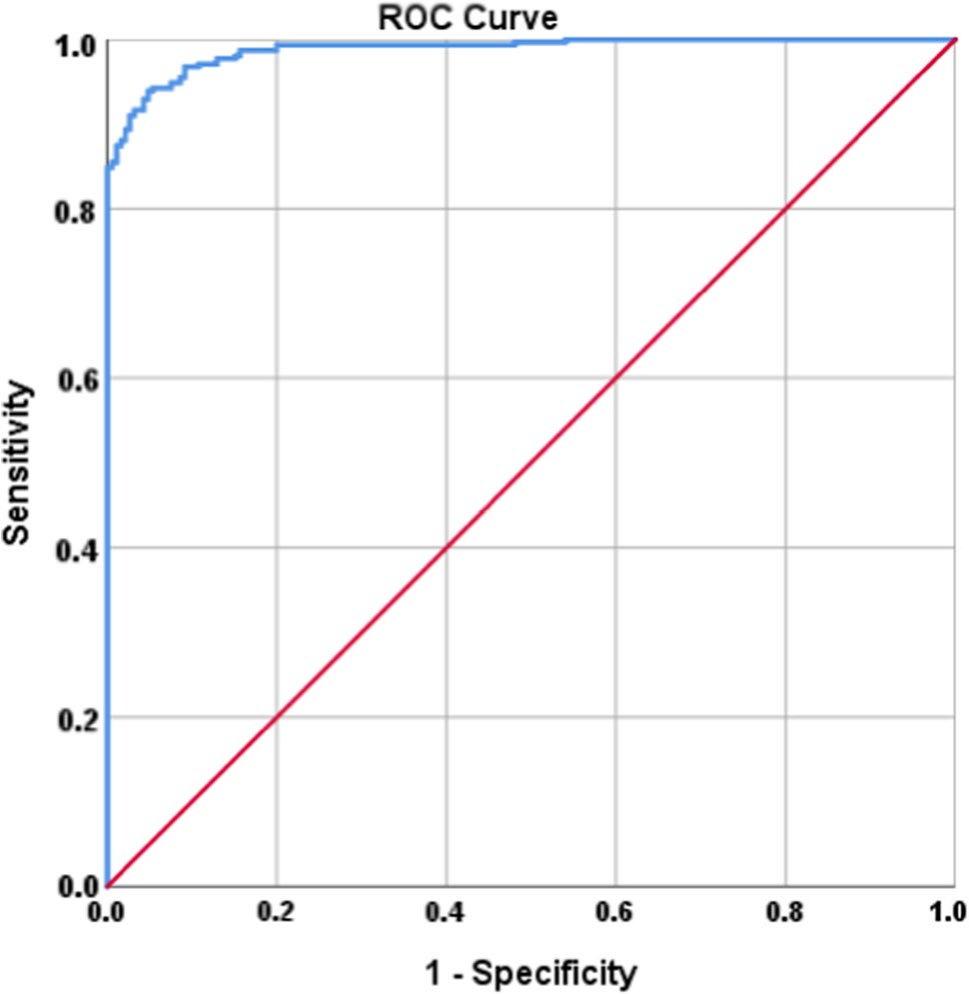
ROC curve to detect the predicted probability of the joint indicators (AISI, CRP and D-dimer) for ICU admission. The area under ROC curve of the combination of these three parameters was 0.98 (*P* < 0.001). *AISI* Aggregate Index of Systemic Inflammation, *CRP* C-reactive protein, *ICU* intensive care unit

As indicated by multivariable analysis, the three biomarkers (AISI, CRP, and D-dimer) were independent predictors for ICU admission. The area under ROC curve of the combination of these three parameters was 0.98. The predictive ability of joint indicators showed the superiority over the single index (*P* < 0.001) (Fig. 4).

## Discussion

COVID-19 is the most lethal pandemic of recent history, with a large spectrum of severity, ranging from mild or even asymptomatic cases to severe presentations requiring ICU admission. COVID-19 infection showed fluctuations in the disease spread, with peaks exceeding half a million cases per day worldwide, so ICU capacities are insufficient to face such increasing demands. Consequently, we used data obtained at the peak of COVID-19 in 2020, aiming to assist in the ICU admission decision depending on easily accessible, rapid, and simple tests such as CBC.

Older patients often have more comorbidities and less immunity, so it is well accepted to have a higher risk for admission in ICU. Like our study, many other studies proved the relation between COVID 19 severity and old age [19–21]. However, a large analysis performed on more than 5000 cases did not detect a significant age difference between ICU and non-ICU patients [22].

In considering comorbidities as DM, HTN, COPD, and CVD, the ICU group in our study had significantly more associated diseases than in previous studies [20, 23]. Medical history of other conditions was a top predictor of mortality in COVID 19 patients than a parameter for ICU entry in other studies [15, 24].

CBC has been widely used to test inflammatory processes and diagnose many diseases [25]. Being simple, inexpensive, and informative investigation, CBC has been extensively investigated in the majority of COVID 19 studies. Higher WBCs, ANC, AMC, and NLR were observed in more critical COVID-19 patients [10, 23, 26], and this is in line with our results. ALC and PLT predominate in less severe cases [10, 20, 24]. Neutrophils are the first step of innate immune defense. They play a crucial defensive role in bacterial and fungal infection by destroying these microorganisms via phagocytosis and creating neutrophil extracellular traps (NET). However, their attribute in viral infections remains unclear. Neutrophils do not seem essential for virus clearance from pulmonary cells and host survival in mice infected by SARS-CoV [27]. The autopsy of patients affected by COVID-19 demonstrated significant neutrophil infiltration in pulmonary capillaries and extravasation into alveolar spaces. The development of both trachea neutrophilic mucositis and acute capillaritis indicates profound inflammation in the airways [27]. Lymphopenia described in COVID-19, on the other hand, seems to be related to the virus’s ability to infect T cells depending on angiotensin-converting enzyme 2 (ACE2) receptors and CD147-spike protein [28].

The combined ratios of these parameters are often used as inflammation indices. These parameters are recommended as biomarkers to assist in diagnosing inflammation, progression, and risk stratification. The NLR, derived NLR (d-NLR), PLR, and monocyte to lymphocyte ratio (MLR) have recently been proved to play a crucial role in diagnosis and severity evaluation of COVID-19 cases [18, 19, 29]; Interestingly, it has been documented that NLR value is a sensitive inflammation marker than absolute neutrophil and lymphocyte levels [28]. However, up to our knowledge, none of the previous research works have assessed the predictive value of SII, NLPR, SIRI, and AISI to assess the need of COVID-19 patients for ICU admission.

ROC curve was used to detect the optimal cut-off values of SII, NLPR, SIRI, and AISI, which revealed that SII and SIRI had the highest areas under the curve, with SII having an optimal cut-off value of 1346 and SIRI had an optimal cut-off value of 2.5. A previous study which investigated the optimal cut-off values of these indexes, detected significant AUC with SIRI (cut-off value 2.9) and AISI (cut-off value 798), while borderline significant AUC with SII [11]. On the contrary, another study revealed NLPR to be the index with the highest AUC (0.7); AUC was in line with those of previous reports, ranging between 0.65 and 0.73 [30–32]. SII, which relies on thrombocytes, neutrophils, and lymphocytes, is a recently proposed score. The SII was suggested as a prognostic marker in sepsis patients’ follow-up, as an index describing the instability in the inflammatory response [32]. Moreover, SII effectively predicts the prognosis of hepatocellular carcinoma and small cell lung cancer [12]. In another interesting study, compared to healthy controls, SII was significantly altered in COVID-19 patients, indicating a diagnostic role in patients infected with SARS-CoV2 [25].

In accordance with a previous study, Kaplan–Meier survival curves using cut-off values obtained from ROC curves showed that survival was significantly related to AISI, NLPR, SII, and SIRI [11]. Kaplan–Meier survival curves showed substantially lower survival in patients with higher AISI, NLPR, SII, and SIRI.

Univariate and multivariate logistic regression was conducted to predict possible factors that increase the need for admission to ICU; among all studied clinically factors, only age, AISI, CRP, D-dimer, and use of oxygen aid were independent early predictors for ICU admission while the role of other combined indexes as NLPR, SI, and SIRI was unclear. According to previous research, AISI was significantly higher in COVID-19 pneumonic patients than in non-pneumonic cases [9]. This coincides with our observations in which AISI was higher in the ICU group than the non-ICU group as all ICU patients were pneumonic, as evidenced by MSCT imaging. In a previous similar study, elevated SII was the independent adverse factor affecting COVID 19 patients’ survival after adjusting for confounders reported in univariate analysis [11].

The limitations of this research included its retrospective design, the complexity of the used indices which may create a difficulty to use them in the practice, and the probability of related confounders, despite attempts to prevent it. Another study limitation is that unlike other biomarkers, there has been no clear consensus on the standard cut-off values of AISI, NLPR, SII, and SIRI. It is unknown whether the cutoffs identified in this study can be applied to other populations.

## Conclusion

According to our results and observations in the current study, AISI appears a reasonable and attractive predictor for admission to ICU. We encourage early calculation of this unique score to sort outpatients of COVID-19 on admission. SII was linked to survival rather than admission to ICU, while the other indexes mostly had an additive role that needed further evaluations. The use of AISI, D-dimer, and CPR together showed a predictive ability higher than the use of a single index.

*AISI* Aggregate Index of Systemic Inflammation, *COPD* chronic obstructive pulmonary disease, *CRP* C-reactive protein, *DM* diabetes mellitus, *HTN* hypertension, *NLPR* neutrophils lymphocyte to platelet ratio,* SII,* systemic immune-inflammation index, *SIRI* systemic inflammation response index

## Supplementary Information

Below is the link to the electronic supplementary material.

Supplementary file1 (DOCX 15 kb)

## Data Availability

The data that support the findings of this study are available from the corresponding author upon reasonable request.

## Abbreviations

AISI: Aggregate Index of Systemic Inflammation
CBC: Complete blood picture
COPD: Chronic obstructive pulmonary disease
CPAP: Continuous positive ventilation pressure
CRP: C-reactive protein
CT: Computed tomography
CVD: Cardiovascular disease
DM: Diabetes mellitus
HTN: Hypertension
ICU: Intensive care unit
NLPR: Neutrophils lymphocyte to platelet ratio
NLR: Neutrophil to lymphocyte ratio
LMR: Lymphocyte to monocyte ratio
PLR: Platelet to lymphocyte ratio
SII: Systemic immune-inflammation index
SIRI: Systemic inflammation response index
WHO: World Health Organization

## References

[1] Rahmandad H, Lim TY, Sterman J. Behavioral dynamics of COVID-19: Estimating under-reporting, multiple waves, and adherence fatigue across 92 nations. 2021;92. 10.1002/sdr.1673 PMC8250772 34230767

[2] Scudellari M (2020). How the pandemic might play out in 2021 and beyond. Nature..

[3] Rodriguez-Morales AJ, Cardona-Ospina JA, Gutiérrez-Ocampo E, Villamizar-Peña R, Holguin-Rivera Y, Escalera-Antezana JP (2020). Clinical, laboratory and imaging features of COVID-19: a systematic review and meta-analysis. Travel Med Infect Dis..

[4] Chi G, Lee JJ, Jamil A, Gunnam V, Najafi H, Memar Montazerin S (2020). Venous thromboembolism among hospitalized patients with COVID-19 undergoing thromboprophylaxis: a systematic review and meta-analysis. J Clin Med.

[5] Yang A-P, Liu J, Tao W, Li H-M (2020). The diagnostic and predictive role of NLR, d-NLR and PLR in COVID-19 patients. Int Immunopharmacol..

[6] Qu R, Ling Y, Zhang YZ, Wei LY, Chen X, Li XM (2020). Platelet-to-lymphocyte ratio is associated with prognosis in patients with coronavirus disease-19. J Med Virol..

[7] Merekoulias G, Alexopoulos EC, Belezos T, Panagiotopoulou E, Jelastopulu E (2010). Lymphocyte to monocyte ratio as a screening tool for influenza. PLoS Currents..

[8] Lee JJ, Montazerin SM, Jamil A, Jamil U, Marszalek J, Chuang ML (2021). Association between red blood cell distribution width and mortality and severity among patients with COVID-19: A systematic review and meta-analysis. J Med Virol.

[9] Paliogiannis P, Putzu C, Cortinovis D, Colonese F, Canova S, Fois A, et al. Blood cell count indexes of systemic inflammation as predictive biomarkers of immunotherapy outcomes in advanced non-small-cell lung cancer 2018. 10.1007/s00262-018-2182-4 PMC11028046 29947960

[10] Paliogiannis P, Zinellu A, Scano V, Mulas G, De Riu G, Pascale RM (2020). Laboratory test alterations in patients with COVID-19 and non COVID-19 interstitial pneumonia: a preliminary report. J Infect Dev Count.

[11] Fois AG, Paliogiannis P, Scano V, Cau S, Babudieri S, Perra R (2020). The systemic inflammation index on admission predicts in-hospital mortality in COVID-19 patients. Molecules.

[12] Hu B, Yang X-R, Xu Y, Sun Y-F, Sun C, Guo W (2014). Systemic immune-inflammation index predicts prognosis of patients after curative resection for hepatocellular carcinoma. Clin Cancer Res.

[13] Li H, Huang J-b, Pan W, Zhang C-t, Chang X-y, Yang B. Systemic Immune-Inflammatory Index predicts prognosis of patients with COVID-19: a retrospective study. 2020.

[14] Furuncuoğlu Y, Tulgar S, Dogan A, Cakar S, Tulgar Y, Cakiroglu B (2016). How obesity affects the neutrophil/lymphocyte and platelet/lymphocyte ratio, systemic immune-inflammatory index and platelet indices: a retrospective study. Eur Rev Med Pharmacol Sci.

[15] Li S, Lan X, Gao H, Li Z, Chen L, Wang W (2017). Systemic Inflammation Response Index (SIRI), cancer stem cells and survival of localised gastric adenocarcinoma after curative resection. J Cancer Res Clin Oncol.

[16] Chen L, Kong X, Wang Z, Wang X, Fang Y, Wang J (2020). Pretreatment systemic inflammation response index in patients with breast cancer treated with neoadjuvant chemotherapy as a useful prognostic indicator. Cancer Manag Res.

[17] Organization WH (2020). Clinical management of severe acute respiratory infection (SARI) when COVID-19 disease is suspected: interim guidance, 13 March 2020.

[18] Liao X, Wang B, Kang Y (2020). Novel coronavirus infection during the 2019–2020 epidemic: preparing intensive care units—the experience in Sichuan Province, China. Intensive Care Med.

[19] Guan W-J, Ni Z-Y, Hu Y, Liang W-H, Ou C-Q, He J-X (2020). Clinical characteristics of coronavirus disease 2019 in China. N Engl J Med.

[20] Zhao Z, Chen A, Hou W, Graham JM, Li H, Richman PS (2020). Prediction model and risk scores of ICU admission and mortality in COVID-19. PLoS ONE.

[21] Zhou F, Yu T, Du R, Fan G, Liu Y, Liu Z (2020). Clinical course and risk factors for mortality of adult inpatients with COVID-19 in Wuhan, China: a retrospective cohort study. The lancet.

[22] Li X, Ge P, Zhu J, Li H, Graham J, Singer A (2020). Deep learning prediction of likelihood of ICU admission and mortality in COVID-19 patients using clinical variables. PeerJ.

[23] Qin C, Zhou L, Hu Z, Zhang S, Yang S, Tao Y (2020). Dysregulation of immune response in patients with COVID-19 in Wuhan. China. Clin Infect Dis..

[24] Huang G, Kovalic AJ, Graber CJ (2020). Prognostic value of leukocytosis and lymphopenia for coronavirus disease severity. Emerg Infect Dis.

[25] Usul E, Şan İ, Bekgöz B, Şahin A (2020). Role of hematological parameters in COVID-19 patients in the emergency room. Biomark Med.

[26] Huang C, Wang Y, Li X, Ren L, Zhao J, Hu Y (2020). Clinical features of patients infected with 2019 novel coronavirus in Wuhan. China The lancet.

[27] Tomar B, Anders H-J, Desai J, Mulay SR (2020). Neutrophils and neutrophil extracellular traps drive necroinflammation in COVID-19. Cells.

[28] Chan AS, Rout A (2020). Use of neutrophil-to-lymphocyte and platelet-to-lymphocyte ratios in COVID-19. J Clin Med Res.

[29] Aly MM, Meshref TS, Abdelhameid MA, Ahmed SA, Shaltout AS, Abdel-Moniem AE (2021). Can hematological ratios predict outcome of COVID-19 patients? a multicentric study. J Blood Med.

[30] Tatum D, Taghavi S, Houghton A, Stover J, Toraih E, Duchesne J (2020). Neutrophil-to-lymphocyte ratio and outcomes in Louisiana Covid-19 patients. Shock (Augusta, Ga)..

[31] Zhang H, Cao X, Kong M, Mao X, Huang L, He P (2020). Clinical and hematological characteristics of 88 patients with COVID-19. Int J Lab Hematol.

[32] Lagunas-Alvarado M, Mijangos-Huesca FJ, Terán-González J, Lagunas-Alvarado MG, Martínez-Zavala N, Reyes-Franco I (2017). Systemic immune inflammatory index in sepsis. Med Intern México.

