# Supplementary material for: Combined Blood Indexes of Systemic Inflammation as a Mirror to Admission to Intensive Care Unit in COVID-19 Patients: A Multicentric Study
Source: J Epidemiol Glob Health. 2021 Dec 13;12(1):64–73. doi: 10.1007/s44197-021-00021-5 (PMC8668150; doi:10.1007/s44197-021-00021-5)
Supplement: Supplementary file 1 — Supplementary file1 (DOCX 15 kb) [file 44197_2021_21_MOESM1_ESM.docx]

**Supplementary Table 1**:Modified National Early Warning Score (ANEWS) for COVID-19*.*

| **Parameters** | **3** | **2** | **1** | **0** | **1** | **2** | **3** |
| --- | --- | --- | --- | --- | --- | --- | --- |
| **Age** |  |  |  | <65 |  | ≥65 |  |
| **Comorbidities (DM, HTN, COPD, CKD, Malignant tumors)** |  |  |  | 0 | 1 | ≥2 |  |
| **Resp. rate (per minute)** | ≤8 |  | 9–11 | 12–20 |  | 21–24 | ≥25 |
| **Oxygen saturation (room air) %** | ≤91 | 92–93 | 94–95 | ≥96 |  |  |  |
| **Oxygen supplement necessary** |  | Yes |  | No |  |  |  |
| **Systolic blood pressure (mmHg)** | ≤90 | 91–100 | 101–110 | 111–219 |  |  | ≥220 |
| **Pulse (per min)** | ≤40 |  | 41–50 | 51–90 | 91–110 | 111–130 | ≥131 |
| **Consciousness** |  |  |  | Alert |  |  | CVPU |
| **Temperature (°C)** | ≤35.0 |  | 35.1–36.0 | 36.1–38.0 | 38.1–39.0 | ≥39.1 |  |

**DM**, diabetes mellitus; **HTN,** hypertension**; COPD,** chronic obstructive pulmonary disease; **CKD,** chronic kidney disease; **CVPU,** confusion, verbal pain unresponsiveness.

**Modified NEWS threshold and triggers**

| Modified NEW score | Clinical risk | response |
| --- | --- | --- |
| **Aggregate score 0-4** | Low | Ward-based response* |
| **Red score**  **Score of 3 in only individual parameter** | Low-medium | Urgent ward-based response* |
| **Aggregate score 5-6** | Medium | Key threshold for urgent response* |
| **Aggregate score 7 or more** | High | Urgent or emergency response** |

*response by a clinician or team with competence in the assessment and treatment of acutely ill patients when the escalation of care to a critical care team is appropriate.

**the response team must also include staff with critical care skills, including airway management.
